# Possible extracardiac predictors of aortic dissection in Marfan syndrome

**DOI:** 10.1186/1471-2261-14-47

**Published:** 2014-04-11

**Authors:** Bence Ágg, Kálmán Benke, Bálint Szilveszter, Miklós Pólos, László Daróczi, Balázs Odler, Zsolt B Nagy, Ferenc Tarr, Béla Merkely, Zoltán Szabolcs

**Affiliations:** 1 Heart and Vascular Center, Semmelweis University, Városmajor str. 68, 1122 Budapest, Hungary; 2 Department of Pulmonology, Semmelweis University, Budapest, Hungary; 3 Association of Genetics for Health, Budapest, Hungary; 4 Hungarian Marfan Foundation, Budapest, Hungary

**Keywords:** Marfan syndrome, Aortic dissection, Predictors, Cardiac surgery

## Abstract

**Background:**

According to previous studies, aortic diameter alone seems to be insufficient to predict the event of aortic dissection in Marfan syndrome (MFS). Determining the optimal schedule for preventive aortic root replacement (ARR) aortic growth rate is of importance, as well as family history, however, none of them appear to be decisive. Thus, the aim of this study was to search for potential predictors of aortic dissection in MFS.

**Methods:**

A Marfan Biobank consisting of 79 MFS patients was established. Thirty-nine MFS patients who underwent ARR were assigned into three groups based on the indication for surgery (dissection, annuloaortic ectasia and prophylactic surgery). The prophylactic surgery group was excluded from the study. Transforming growth factor-β (TGF-β) serum levels were measured by ELISA, relative expression of c-Fos, matrix metalloproteinase 3 and 9 (MMP-3 and −9) were assessed by RT-PCR. Clinical parameters, including anthropometric variables - based on the original Ghent criteria were also analyzed.

**Results:**

Among patients with aortic dissection, TGF-β serum level was elevated (43.78 ± 6.51 vs. 31.64 ± 4.99 ng/l, p < 0.0001), MMP-3 was up-regulated (Ln2^α^ = 1.87, p = 0.062) and striae atrophicae were more common (92% vs. 41% p = 0.027) compared to the annuloaortic ectasia group.

**Conclusions:**

We found three easily measurable parameters (striae atrophicae, TGF-β serum level, MMP-3) that may help to predict the risk of aortic dissection in MFS. Based on these findings a new classification of MFS, that is benign or malignant is also proposed, which could be taken into consideration in determining the timing of prophylactic ARR.

## Background

Marfan syndrome is an autosomal dominantly inherited genetic disorder affecting the body’s connective tissue. The prevalence of the syndrome is 1:3000–1:5000 [1]. Among others, it involves the cardiovascular system, eyes and the skeletal system. Most patients with Marfan syndrome were shown to have a mutation in the fibrillin-1 (FBN1) gene [1,2]. The mutation causes decreased Large Latent Complex (LLC) sequestering ability of the FBN1 protein, which in turn, leads to elevated tissue and serum concentrations of Transforming Growth Factor-β (TGF-β). Elevated TGF-β affects the expression of many proteins in the connective tissue through both the standard Smad mediated signal transduction, and alternative (non-canonical) pathways including MAP kinases (ERK1/2, JNK1, p38) with AP-1 transcription factor (c-Fos/c-Jun complex) as a downstream mediator [3]. High tissue and plasma activity of matrix metalloproteinases (MMPs) – especially gelatinases (MMP-2 and 9) and MMP-3 – are prominent [4-6]. These molecular alterations entail the fragmentation of the connective tissue fibres, and results in an inelastic and tear-prone connective tissue. Due to this inelasticity the wall of the aorta is not able to resist the force arising from blood pressure, leading to the formation of aortic pathologies typical to the syndrome [6,7].

According to Détaint and colleagues by the age of 60 in nearly 100 percent of the individuals with Marfan syndrome, varying degrees of aortic root dilatation will have been developed and three quarters of the patients would have undergone aortic root replacement (ARR*),* as the indication criteria for surgery are fulfilled: either increased aortic diameter to critical level, symptomatic aortic valve insufficiency or Stanford type “A” dissection [8]. In most of the Marfan cases, aortic dissection presents an acute life-threatening condition, and is to be treated by surgery on an emergency basis. Consequently, prophylactic ARR should be given priority in Marfan syndrome patients’ care, in order to prevent aortic dissection.

Optimal timing of these properly planned operations with less potential of complications, has a decisive importance with regard to life expectancy and quality of life of Marfan patients [9]. However, cardiac surgical interventions performed unreasonably early (with small aortic diameters) in young adulthood or childhood may lead to early and late complications, in certain cases leading to reoperation or redo surgery [10,11]. Moreover, in cases with mechanical valve implantation lifelong anti-coagulation is unavoidable [12]. These factors can significantly worsen the quality of life of the asymptomatic young patients. In addition, numerous studies have shown that aortic dissection can also develop in individuals with smaller aortic diameters compared to those dimensions, which are included in the current surgical indication criteria [7,13]. These data suggest, that aortic diameter alone is not an appropriate indicator of aortic dissection.

To optimize the timing of the preventive surgical intervention, it would be necessary to create a model that could precisely determine the risk and also the probable onset of aortic dissection for each individual. Hence, we established a systematic Marfan Biobank with relatively large sample collection, in order to gain a better overview of pathomechanism and the underlying molecular pathways of the diverse cardiovascular manifestations. Based on the Biobank data, we present three possible extracardiac predictors of aortic dissection in Marfan syndrome, and we propose a new classification (benign and malignant form) indicating the probable cardiovascular outcome.

## Methods

Patients’ selection was implemented from the Hungarian Marfan Registry (HMR) set up and maintained by the staff of the Heart and Vascular Center at Semmelweis University. Clinical data of 221 Hungarian patients with Marfan syndrome were collected in the HMR between 1988 and 2011. In each patient enrolled in the HMR, diagnosis was established according to the original Ghent nosology [14]. However in 2011, when patients were selected for the current study, the diagnosis was verified in every case with the use of the revised (2010) Ghent criteria [15]. The ethical approval was obtained from the Scientific and Research Ethical Committee of the Medical Research Council (ETT-TUKEB 13699/2011).

### Ghent criteria

The main components of the original Ghent nosology were the major and minor criteria representing the degree to which each of the six affected organ systems were involved. According to this classification three further genetic and family history criteria should have been taken into consideration (the presence of FBN1 mutation causing MFS; a haplotype around the FBN1 gene which was also present in a diagnosed MFS relative; parent, child or sibling meeting the diagnostic criteria). If two major criteria in two separate organ systems and the involvement of a third organ system was present, or the patient had a FBN1 mutation proven to cause MFS accompanied by one major criterion and the involvement of another organ system the diagnosis of MFS could have been established in the index case. For relatives of the index case one major criterion and the involvement of another organ system was enough for the diagnosis.

The revised Ghent nosology introduces the concept of the systemic score and outlines seven scenarios for the diagnosis of MFS based on the different combinations of cardinal manifestations (aortic involvement, ectopia lentis, FBN1 mutation, systemic score) and family history. (Tables 1 and 2)

**Table 1:** Original and revised Ghent nosology

**Organ system (involvement)**	**Major**	**Minor**
**Skeletal**	The presence of more than 4:	moderate pectus excavatum
(2 maj OR 1 maj and 2 min)	- pectus carinatum	joint hypermobility
	- pectus excavatum requiring surgical treatment	highly arched palate crowding of teeth
	- reduced USLS ratio or ASHR greater than 1.05	Facial abnormalities:
	- wrist and thumb signs	- dolichocephaly
	- scoliosis of more than 20° or spondylolisthesis	- malar hypoplasia
	- reduced extension of the elbows	- enophtalmus
	- medial displacement of the medial malleolus, pes planus	- retrognathia
	- protusio acetabuli of any degree	- down-slanting palpebral fissures
**Ocular**	ectopia lentis	abnormally flat cornea
(2 min)		increased axial length of the eyeball
		hypoplastic iris, ciliary muscle, decreased miosis
**Cardiovascular**	ascending aortic dilatation	mitral valve prolapse
(1 min OR 1 maj)	dissection of the ascending aorta	pulmonary arteria dilatation
		mitral annulus calcification
		type B aortic dissection <50 years
**Pulmonary** (1 min)		spontaneous pneumothorax
		apical pulmonary blebs
**Skin** (1 min)		striae atrophicae
**Dura** (1 maj)	Lumbosacral dural ectasia	

**Original Ghent nosology** (maj: major criterion/criteria, min: minor criterion/criteria, ASHR: arm span to height ratio, USLS: upper segment to lower segment ratio).

**Table 2:** Revised Ghent nosology

**P**	**Systemic involvement (>7 point)**		**Diagnostic scenarios**	
3	OR	Wrist AND thumb sign	I	Aortic involvement AND Ectopia lentis
1		Wrist OR thumb sign	II	Aortic involvement AND FBN1 mutation
2	OR	Pectus carinatum	III	Aortic involvement AND Systemic involvement
1		Pectus excavatum OR chest asymmetry	IV	Aortic involvement AND Family history
2	OR	Hindfoot deformity	V	Ectopia lentis AND FBN1 mutation
1		Plain pes planus	VI	Ectopia lentis AND Family history
2		Pneumothorax	VII	Systemic involvement AND Family history
2		Dural ectasia		
2		Protusio acetabuli		
1		USLS↓ AND ASHR↑ AND no scoliosis		
1		Scoliosis OR thoracolumbar kyphosis		
1		Reduced elbow extension		
1		Facial abnormalitis (see above)		
1		Striae atrophicae		
1		Myopia > 3 diopters		
1		Mitral valve prolapse		

**Revised Ghent nosology** (P: systemic score point; ASHR: arm span to height ratio, USLS: upper segment to lower segment ratio, FBN1: fibrillin-1 gene).

### Establishment of the Marfan Biobank

63 patients from the HMR were chosen randomly. First and second degree relatives of these 63 patients, who were diagnosed with Marfan syndrome and had a record in HMR, were also assigned to this group (another 16 patients). Thus a total of 79 patients were enrolled in the Marfan Biobank. Between October 2011 and April 2012 clinical data and biological samples (serum, blood, genomic DNA, cDNA from PBMC) from 37 women (47%) and 42 men (53%) with Marfan syndrome were collected in the biobank. At the time of the establishment of the biobank the patients’ average age was 33.2 ± 13.5 years.

Forty out of the 79 patients underwent cardiac surgery (primary type of the surgery were ARR in 39 cases and mitral valve replacement in 1 case). Among the 39 patients with ARR surgical indication was aortic dissection in 12 cases (Group 1; no distinction between the 9 acute and 3 chronic cases was applied in this study), annuloaortic ectasia in 12 cases (Group 2) and prophylactic surgery in 15 cases. Annuloaortic ectasia was defined as the presence of dilated aortic annulus accompanied by severe, hemodynamically significant (grade III-IV) aortic insufficiency. In contrast, ARRs performed in patients, who had an aortic diameter over 40–50 mm – measured at the level of the sinus of Valsalva – or an aortic growth rate exceeding 5 mm/year, were classified as prophylactic surgery, if aortic regurgitation was either mild (grade I-II) or completely absent. The above range of aortic diameters corresponds to the changes in the recommended indication criteria for prophylactic ARR in Marfan syndrome patients published in the past two decades [13,16,17].

It is also to emphasize, that while aortic dissection and annuloaortic ectasia are well-defined, distinct manifestations of Marfan syndrome, patients who underwent prophylactic surgery could have developed either aortic dissection or annuloaortic ectasia without the preventive ARR. Therefore we excluded this mixed, intermediate population from our investigation.

Sample collection was conducted 6.7 ± 5.1 years after the surgery. The samples required were collected and handled according to the international protocol at the Heart and Vascular Center of Semmelweis University. The samples were processed in 2 hours following the collection. Oral mucosa samples were collected from 5 subjects under 14 and their extracted genomic DNA was stored.

### Control group

To avoid variances arising from the patients’ individual biological features, molecular sampling and measurements were conducted on control patients as well. Between October 2010 and December 2011 control biological samples (serum, blood, genomic DNA, cDNA from PBMC) from 33 women (41%) and 47 men (59%) were collected. At the time of sampling, the patients’ average age was 35.4 ± 11.2 years. In control patients’ history, there were no cardiovascular disease, cancer, immune-mediated disease, vascular or cardiac surgical intervention.

### TGFβ1 ELISA

For the measurement of serum active TGF-β1 levels a solid phase ELISA was used, which was designed to measure biologically active TGF-β1 (R&D Systems, Minneapolis, USA). 0.25 ml Serum samples were acidified to a pH of 2–3 with 50 μl 1 N HCL for 10 minutes to activate latent TGF-β, and then re-neutralized to pH 7–8 with 50 μl 1.2 N NaOH/0.5 M HEPES. Results were expressed in ng/ml.

### cDNA synthesis and real-time PCR assay

PBMC was isolated from 5 ml of the anticoagulated blood samples (containing EDTA) of the 76 adult patients. Total RNA from PBMC cells content was isolated with Quick RNA microprep kit (Zymo Research, Irvine, USA). RNA concentration was determined using NanoDrop spectophotometer (NanoDrop, Wilmington, USA). Reverse transcription was performed in 20 μl target volume using 5 μg whole RNS, 75 pmol random hexamer primer, 10 mM dNTP (Invitrogen, Bedford, USA), 20 U M-MuLV Reverse Transciptase enzyme (MBI Fermentas, New York, USA) and 1x-es buffer (MBI Fermentas, New York, USA). The reaction mix was incubated for 2 hours at 42°C. The reverse transcriptase reaction solution was diluted three-fold with nuclease-free water. For the real-time PCR assay, 1 μl diluted cDNS (approximately 15 ng RNA-equivalent) and 1 x SYBR Green Master Mixet (Applied Biosystems, Bedford, USA) were used. Primers were designed using Primer Express Software (Applied Biosystems, Bedford, USA). Primer sequences are detailed in Additional file 1: Table S1. Real-time PCR was performed in 20 μl target volume using 1 μl cDNA, 1 pmol, gene-specific Forward and Reverse primer and 1 x SYBR Green PCR Master mix. All real-time PCR were performed using the MX3000 Real-time PCR (Stratagen, Santa Clara, USA) system with the following settings: 40 cycles at 95°C, denaturing process for 15 seconds, annealing at 60°C, chain elongation and detection for 60 seconds. For each gene, relative expression was normalized using the human *GAPDH* gene as standard.

ΔCt values were calculated as Ct(target gene) - Ct(GAPDH endogenous control gene) in the same sample, while ά value was defined as ΔCt(control sample) - ΔCt(target sample). This value was used to calculate expression fold value (Ln 2ά) by the equation. Significantly up- or down-regulated if the Ln ratio of the ά value is >1 or < −1, respectively, with p < 0.05. The ELISA and real-time assays were validated.

### Clinical variables

In the two specified subgroups we assessed sex ratio, family history, age, anthropometric parameters, presence of major and minor criteria in the original Ghent nosology whereas the systemic score was calculated on the basis of the revised Ghent nosology from 2010 [14,15].

Positive family history was defined as the presence of at least one first- or second-degree relative with the diagnosis of Marfan-syndrome. In cases where the above condition was not met, family history was considered negative (sporadic cases).

Measurement (height, lower segment - LS, arm span - AS, weight) and calculation (upper segment - US, upper segment to lower segment ratio - USLS, arm span to height ratio - ASHR) of anthropometric values were carried out according to the instructions of Ghent nosology [14]. Length parameters and weight were expressed in centimetres and kilograms respectively. Shoe sizes were assessed in the units of continental European system. We used Mosteller’s formula to calculate body surface area (BSA) and the well-known formula for body mass index (BMI).

Along with the collection of biological samples the presence of major and minor Ghent criteria – queried from HMR database – were verified with the use of collected cardiological, ophthalmological, orthopedic and dermatological evidences and inspection.

### Statistical methods

In case of continuous parameters with normal distribution Student’t-test was performed. Non-normally distributed variables were compared with the use of Mann–Whitney *U* test. In case of dichotomous parameters – with regards to the small sample size – two-tailed Fisher’s exact test was calculated. Multivariate logistic regression analysis was performed to identify the independent predictors of aortic dissection. The internationally accepted probability (p < 0.05) was chosen for significance level. Data were stored in Microsoft Excel 2010 and analysed with the SPSS statistical program (version 20.0, Chicago, IL, USA).

## Results

### Molecular biological parameters

**Figure 1:**
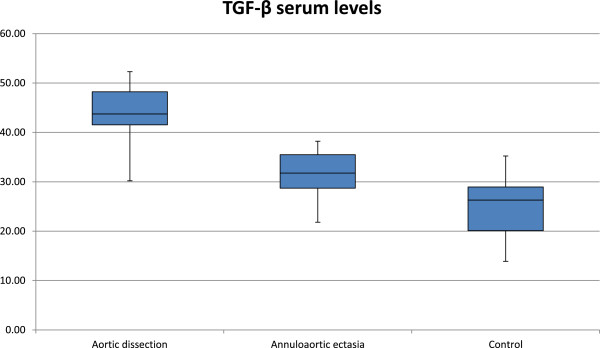
TGF-β serum levels in the aortic dissection, annuloaortic ectasia and in the control patients expressed in ng/ml.

TGF-β serum level was significantly higher (see values below) in both groups established according to the surgical indication, than in the control group (25.7 ± 4.1 ng/l; p < 0.0001 for both groups) (Figure 1).

TGF-β level measured in the dissection (43.78 ± 6.51 ng/l) group was significantly higher than that measured in the annuloaortic ectasia group (31.64 ± 4.99 ng/l; p < 0.0001).

**Figure 2:**
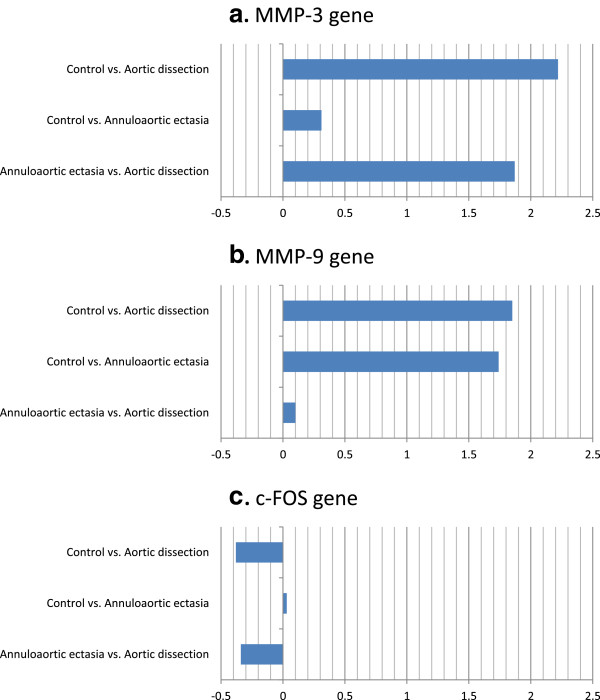
**Ln2**^**α **^**values describing the relative expression of the MMP-3 (a), MMP-9 (b) and c-FOS (c) genes.** ΔCt is Ct(target gene) - Ct(GAPDH endogenous control gene) in the same sample, ά value is ΔCt(control sample) - ΔCt(target sample). This value is used to calculate expression fold value by the equation (expression fold value Ln 2ά). Significantly up- or down-regulated if the Ln ratio of the ά value is >1 or < −1, respectively, with p < 0.05.

The MMP-3 gene showed significant up-regulation in dissection patients with Marfan syndrome in comparison with control patients (Ln2^α^ = 2.22, p = 0.046). There was no change in the MMP-3 gene expression level of the annuloaortic ectasia group when compared to the control group (Ln2^α^ = 0.31, p = 0.089) (Figure 2).

The MMP-3 gene showed up-regulation in dissection patients when compared to annuloaortic ectasia individuals (Ln2^α^ = 1.87, p = 0.062) (Figure 2).

MMP-9 gene showed significant up-regulation in dissection patients compared to the control patients (Ln2^α^  = 1.85, p = 0.043). MMP-9 gene showed up-regulation in annuloaortic ectasia patients compared to the control patients, however, this change was insignificant (Ln2^α^ = 1.74, p = 0.069) (Figure 2).

Compared to the annuloaortic ectasia patients there was no significant difference in the MMP-9 gene expression level of dissection patients with Marfan syndrome (Ln2^α^ = 0.11, p = 0.039) (Table 3).

**Table 3:** Gene expression

	**MMP-3**			**MMP-9**			**c-FOS**		
**Compared groups**	**α value ± SE (α)**	**Ln 2**^**α**^	**p**	**α value ± SE (α)**	**Ln 2**^**α**^	**p**	**α value ± SE (α)**	**Ln 2**^**α**^	**p**
C vs 1	3.21 ± 0.95 (α1)	2.22	0.046*	2.68 ± 0.84 (α1)	1.85	0.043*	−0.56 ± 1.03 (α1)	−0.38	0.096
C vs 2	0.46 ± 1.02 (α2)	0.31	0.089	2.52 ± 1.03 (α2)	1.74	0.069	0.05 ± 0.99 (α2)	0.03	0.081
2 vs 1	2.7 ± 0.91 (α3)	1.87	0.062	0.16 ± 0.52 (α3)	0.11	0.039*	−0.5 ± 0.62 (α3)	−0.34	0.083

*significant data, α1 = ΔCtC– ΔCt1, α2 = ΔCtC – ΔCt2, α3 = ΔCt2 – ΔCt1; 1: Aortic dissection group; 2: Annuloaortic ectasia group; C: Control group.

In the expression of the c-FOS gene there was no difference between the dissection and annuloaortic ectasia groups compared to the control group (Ln2^α^ = −0.38, p = 0.096; Ln2^α^ = 0.03, p = 0.081 respectively). Investigating the subgroups established according to surgical indication, there was no difference between them in the c-FOS gene expression (dissection vs. annuloaortic ectasia: Ln2^α^ = −0.34, p = 0.083) (Figure 2), (Table 3).

### Clinical variables

Our findings from the analysis of clinical parameters are illustrated in Table 4.

**Table 4:** Clinical data

	**Aortic dissection**	**Annuloaortic ectasia**	**p**
**Patients**	12	12	
**Anthropometric (measured)**			
Height	178.5 ± 10.30	186.2 ± 11.74	0.106
Lower segment (cm)	93.58 ± 9.45	101.9 ± 10.95	0.058
Arm span (cm)	186.92 ± 13.78	189.6 ± 15.2	0.656
Foot size	42.2 ± 2.5	44.0 ± 3.1	0.148
Weight (kg)	74.9 ± 14.7	75.0 ± 17.0	0.989
**Anthropometric (calculated)**			
Upper segment (cm)	85.0 ± 7.8	84.2 ± 9.9	0.839
Body Mass Index (BMI; kg/m^2^)	23.4 ± 3.70	21.45 ± 3.62	0.204
Body surface area (m^2^)	1.92 ± 0.22	1.961 ± 0.27	0.686
Upper segment - Lower segment ratio (USLS)	0.918 ± 0.132	0.838 ± 0.147	0.176
Arm span - Height ratio (ASHR)	1.046 ± 0.042	1.018 ± 0.039	0.092
**TGF-β serum level (ng/l)**	43.78 ± 6.51	31.64 ± 4.99	<0.0001^*^
**Ghent nosology (%)**			
Mitral valve prolapse	58%	75%	0.667
Pectus carinatum	33%	67%	0.220
Pectus excavatum requiring surgery	17%	8%	1.000
Reduced upper to lower segment ratio	33%	50%	0.680
Increased arm span to height ratio	58%	17%	0.089
Wrist sign	83%	83%	1.000
Thumb sign	83%	83%	1.000
Scoliosis of > 20° or spondylolisthesis	83%	58%	0.370
Severe scoliosis	58%	42%	0.684
Reduced extension at the elbows	17%	8%	1.000
Medial displacement of the medial malleolus causing pes planus	33%	33%	1.000
Heel deformity	8%	17%	1.000
Pectus excavatum of moderate severity	25%	33%	1.000
Asymetric chest	33%	33%	1.000
Joint hypermobility	75%	42%	0.213
Highly arched palate with crowding of teeth	58%	42%	0.684
Facial appearance	50%	16%	0.400
Dolichocephaly	25%	17%	1.000
Enophtalmos	8%	8%	1.000
Retrognathia	42%	8%	0.155
Ectopia lentis	25%	17%	1.000
Myopia over 3 diopter	8%	17%	1.000
Increased axial length of globe	0%	8%	1.000
Spontaneous pneumothorax	8%	8%	1.000
Striae atrophicae (stretch marks)	92%	42%	0.027^*^
**Positive family history (%)**	50%	50%	1.000
**Sex (male)**	4	9	0.099
**Systemic score**	7 [5-8]	7 [5.5-8]	0.860

*significant data.

These results highlight the fact, that the presence of one criteria from the Ghent nostology, namely striae atrophicae, was significantly more common among dissection patients than in the annuloaortic ectasia group (92% vs. 41% p = 0.027) (Table 4).

According to the low number of observed Marfan patients, only TGF-β revealed as an independent risk factor for aortic dissection (OR 1.609, 95% CI 1.068-2.424, p = 0.023).

## Discussion

The key issue in the treatment of patients with Marfan syndrome is the prevention of life-threatening aortic dissection for which - according to the current state of knowledge - the only solution seems to be ARR. The increasing risk of acute aortic dissection caused by progressive aortic dilatation can only be counterbalanced by preventive cardiac surgery, performed at small aortic diameters at the early stages of the disease [18,19]. Furthermore, the short and long-term survival rates for patients having undergone prophylactic surgery, as well as the rates of complications and subsequent reoperations are far more favourable than for those, who have ARR procedures in an emergency situation [9,17]. In the past two decades ARR surgery tends to be performed at smaller and smaller aortic diameter (in 1991 at >6 cm [16]; in 1994 at >5.5 cm [17]; in 2005 at >4.5 cm [13]; nowadays certain experts recommend it at even >3 cm [16]). Thus, because of the progressive nature of aortic dilatation, patients are much younger nowadays when surgical intervention is performed.

However, ARR performed in a young patient – as any surgical treatment – may have considerable risks. After ARR procedure, several life-threatening complications may occur even among patients having no cardiac symptoms prior to the operation [10-12].

It has also been pointed out that in case of Marfan syndrome, aortic dissection may occur at smaller aortic diameters, even smaller than those, quoted in the latest surgical indication criteria [7,13]. Thus, considering literature data presented above, aortic diameter, alone does not seem to be sufficient and satisfactory to predict the formation of aortic dissection.

### New classification: benign and malignant form

In order to rule out certain limitations, several attempts have been made. To exclude the strong variations in body size typical to Marfan syndrome patients, Aalberts et al. specified *aortic diameters* normalized to body surface area and patient age, instead of using the absolute value [20]. In addition, patients’ *family history* of aortic dissection as well as *aortic growth rate* is taken into account by most of the protocols [20,21]. Nevertheless, besides these factors, identification of other clinical and molecular markers would be necessary, to predict the trends describing the progression and direction of the aortic pathology.

Being aware of the prognostic parameters (both clinical and molecular biological) of aortic dissection, preventive surgical intervention – which usually exposes Marfan syndrome patients to considerable risks and potential complications – should only be carried out, if there is a real danger of aortic dissection. Optimal timing for ARR procedure could be also calculated more precisely, which would further improve the life expectancy and quality of life of patients with Marfan syndrome.

As Meijboom et al. showed in their research, the growth rate of aortic dilatation is quite variable among Marfan syndrome patients [13]. At early stages, when aortic growth rate is slow (“slow grower”) the disease is asymptomatic. However, during the decades, patients develop annuloaortic ectasia, which leads to aortic valve insufficiency. In this case, the risk of dissection is lower. In contrast, rapid growth of the aortic root (“fast grower”) can be characterized by an increased risk of aortic dissection.

Based on the data above, patients with Marfan syndrome should be assigned into two groups: *malignant* (with an increased risk of dissection) and *benign* (with a reduced risk of dissection). However, instead of the growth rate alone as Meijboom et al. suggested, we hypothesize that the main difference between the two groups lies in the presence or absence of certain, yet unmapped, pathological alterations at molecular biological and connecting tissue level, which can cause aortic dissection even in case of aortic diameters close to normal [7,13]. We also assume that these molecular alterations, if present in a patient, can also influence the pattern of the extracardiac manifestations of the syndrome.

### Molecular and extracardiac phenotypic characteristics of the hypothetical malignant group

It is known that the tissue and serum levels of TGF-β, which increase the expression of several proteolytic enzymes, are elevated in patients with Marfan syndrome [6,22]. However, our results suggest, that molecular alteration of this kind is more pronounced in dissection patients. The highly significant difference in the TGF-β serum level between the dissection and annuloaortic ectasia groups indicates, that increased TGF-β levels may not only play an important role in the development of the symptoms, but also the degree of the increase in the serum levels may correlate with the risk of dissection.

Furthermore, increased MMP-3 expression in the PBMC cells of the patients with dissection compared to the annuloaortic ectasia group, apparently contributes to the development of the severe pathologic changes leading to aortic dissection. Since in terms of c-FOS expression no distinction could be identified between these two groups, it seems unlikely that the detrimental effects of increased TGF-β level are resulting from the activation of the ERK1/2, JNK1, p38 mediated non-canonical pathways and the consequent up-regulation of the AP-1 transcription factor (AP-1 is a heterodimer of c-Fos and other proteins). Thus, it is more likely that Smad mediated canonical or Rho-like GTPase and PI3K/Akt mediated non-canonical pathways are responsible for the signal transduction causing the up-regulation of MMP-3 and destruction of the aortic wall [4].

**Figure 3:**
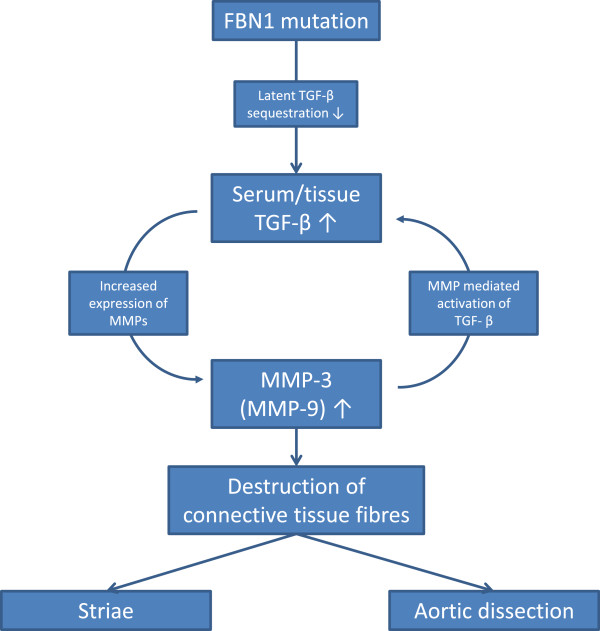
**This figure depicts a hypothetical positive feedback cycle in the homeostasis of connective tissue fibres.** As shown in the picture fibrillin-1 mutation leads to elevated serum and tissue Transforming Growth Factor-β levels, as the ability of fibrillin-1 protein to sequester latent TGF-β decreases. Through intracellular signal transduction active TGF-β up-regulates matrix metalloproteinases (MMP-3 and MMP-9). Increased MMP activity in the connective tissue in turn leads to the integrin dependent activation of latent TGF-β which results in further increase of the active TGF-β tissue level. Both aortic dissection and striae (stretch marks) may be the consequence of increased tissue activity of MMPs. This positive feedback cycle could provide a link between aortic dissection and the three extracardiac predictors (TGF-β serum level, MMP-3 expression and striae atrophicae) described in this article.

Results from an earlier study show that MMP-3 is also responsible for the activation of latent TGF-β; therefore, at this central point of the signaling pathway a positive feedback cycle should be considered [6,23] (Figure 3).

It should be stated, that sample collection happened 7.1 ± 5.1 years after the event of aortic dissection, thus it can be ruled out that the above mentioned molecular alterations (elevated TGF-β levels, up-regulated MMP-3) is caused by the dissection itself.

Our findings also indicate that certain groups of patients with Marfan syndrome can be characterized by yet unidentified phenotypic correlations. The development of aortic dissection is significantly more often associated with atrophic striae compared to annuloaortic ectasia.

There are limited data available on the molecular mechanisms causing atrophic striae. Although, based on previous publications and on our gene expression results, we assume that the increased MMP-3 and MMP-9 activity in dissection patients is responsible for accelerated degradation of the connective tissue fibres [4,5,24]. Besides the aortic wall, this destruction process may affect the dermis and the subcutaneous layer of the skin as well. Therefore MMP-3 and MMP-9 can be considered as common mediators in the pathogenesis of both aortic dissection and atrophic striae (Figure 3).

In summary, increased presence of atrophic striae along with the above mentioned two molecular markers (elevated TGF-β levels and MMP-3 up-regulation) may be important characteristics of the hypothetical malignant group.

## Conclusions

Based on our findings we conclude that our hypothetical (dignity) groups (benign vs. malignant) are distinct not only from the cardiovascular perspective, but also in terms of phenotypic signs related to other organs (striae), which can be caused by molecular alterations, demonstrated above (TGF-β, MMP-3). Thus, a total of three *–* two molecular and one clinical – factors which might predict aortic dissection were found: elevated TGF-β serum level (1), increased expression of MMP-3 gene in peripheral blood mononuclear cells (2), and the presence of striae atrophicae (3). Differences in the extracardiac clinical parameters can be detected quite easily by inspection (striae), while molecular factors could be assessed by relatively inexpensive laboratory tests (ELISA, RT-PCR).

Similar phenotypic features of the skin - which may predict the cardiovascular pathologies - have not been described in the international literature of Marfan syndrome so far. Likewise, there are also few studies in which the connection between phenotypic differences is analysed [25].

### Limitations, future objectives

Our current findings are not sufficient to define how many of these three predictive factors are necessary to assign a Marfan syndrome patient to the benign or the malignant group; neither can we specify the optimal timing of prophylactic ARR procedure in the malignant or benign group.

In order to prove our hypothesis regarding to the classification of patients with Marfan syndrome into benign and malignant groups, we consider it necessary to repeat the examinations involving a larger number of patients, as well as to carry out further serum level measurements, genetic and gene expression tests. Investigation of molecular pathways underlying certain phenotypic relationships would provide further evidence for the existence of the above mentioned benign-malignant groups and would support their characterization.

According to the low number of observed Marfan patients, only TGF-β revealed as an independent risk factor for aortic dissection. However this study was a pilot study, further examination and the increase of the patient population would be necessary for correct risk scores, which would help to choose the optimal timing of the prophylactic ARR procedure.

### Ethical approval

Medical Research Council ETT-TUKEB 13699/2011.

## Abbreviations

ARR: Aortic root replacement; ASHR: Arm span to height ratio; BMI: Body mass index; BSA: Body surface area; cDNA: Complementary DNA; c-Fos: Cellular oncogene Fos (FBJ murine osteosarcoma viral oncogene homolog); DNA: Deoxyribonucleic acid; ELISA: Enzyme linked immunosorbent assay; FBN1: Fibrillin-1; HMR: Hungarian Marfan Registry; LLC: Large latent complex; MAD: Mothers against decapentaplegic; MAP kinase: Mitogen-activated protein kinase; MMP: Matrix metalloproteinase; PBMC: Peripheral blood mononuclear cell; RT-PCR: Real time – polymerase chain reaction; Sma: Small mutant Caenorhabditis elegans gene; Smad: Sma + MAD; TGF-β: Transforming growth factor-β.

## Competing interests

The authors declare that they have no competing interests.

## Authors’ contributions

BÁ carried out the collection of the samples, participated in the analysis and interpretation of clinical data, drafted the manuscript and performed the statistical analysis. KB carried out the measurements, participated in the analysis and interpretation of clinical data, drafted the manuscript and performed the statistical analysis. BSZ carried out the collection of the samples, participated in the analysis of the molecular biological data. MP participated in the collection of the data, drafted the manuscript and revised the intellectual content. LD participated in the collection of the data, drafted the manuscript and performed the statistical analysis. BO participated in pulmonological clinical examinations. ZSN participated in the ELISA process and in RT-PCR measurements. FT participated in the design of the study and revised the intellectual content. BM revised the manuscript and provided professional advices and institutional background. ZSZ conceived of the study, participated in the coordination of the patients and in the study design, and helped to draft the manuscript. All authors read and approved the final manuscript.

## Pre-publication history

The pre-publication history for this paper can be accessed here:

http://www.biomedcentral.com/1471-2261/14/47/prepub

## Supplementary Material

Additional file 1: Table S1 Gene and primer sequences in this study. Click here for file
