# Supplementary material for: Possible extracardiac predictors of aortic dissection in Marfan syndrome
Source: BMC Cardiovasc Disord. 2014 Apr 11;14:47. doi: 10.1186/1471-2261-14-47 (PMC4021409; doi:10.1186/1471-2261-14-47)
Supplement: Additional file 1: Table S1 — Gene and primer sequences in this study. [file 1471-2261-14-47-S1.doc]

***Table.*** *Gene and primer sequences in this study*

| **Gene name and accession number** | **Forward primer** | **Reverse primer** |
| --- | --- | --- |
| **c-FOS**  (NM_005252) | 5’-GAGAGCTGGTAGTTAGTAGCATGTTGA-3’ | 5’-AATTCCAATAATGAACCCAATAGATTAGTTA-3’ |
| **MMP-3**  (NM_002422) | 5’-GAAATTGGCCACTCCCTGGGT-3’ | 5’-CCTCTGATGGCCCAGAATTGA-3’ |
| **MMP-9**  (NM_004994) | 5’- ACACCGACGACCGGTTTGGC -3’ | 5’-TCCGTGGTGCAGGCGGAGTA-3’ |
